# Social autopsy of neonatal mortality suggests needed improvements in maternal and neonatal interventions in Balaka and Salima districts of Malawi

**DOI:** 10.7189/jogh.05.010416

**Published:** 2015-06

**Authors:** Alain K. Koffi, Tiope Mleme, Humphreys Nsona, Benjamin Banda, Agbessi Amouzou, Henry D. Kalter

**Affiliations:** 1 Department of International Health, Johns Hopkins School of Hygiene and Public Health, Baltimore, MD, USA; 2 National Statistics Office, Zomba, Malawi; 3 Ministry of Health, Blantyre, Malawi; 4 National Statistics Office, Lilongwe, Malawi; 5 UNICEF, New York, NY, USA

## Abstract

**Background:**

The Every Newborn Action Plan calls for reducing the neonatal mortality rates to fewer than 10 deaths per 1000 live births in all countries by 2035. The current study aims to increase our understanding of the social and modifiable factors that can be addressed or reinforced to improve and accelerate the decline in neonatal mortality in Malawi.

**Methods:**

The data come from the 2013 Verbal and Social Autopsy (VASA) study that collected data in order to describe the biological causes and the social determinants of deaths of children under 5 years of age in Balaka and Salima districts of Malawi. This paper analyses the social autopsy data of the neonatal deaths and presents results of a review of the coverage of key interventions along the continuum of normal maternal and newborn care and the description of breakdowns in the care provided for neonatal illnesses within the Pathway to Survival framework.

**Results:**

A total of 320 neonatal deaths were confirmed from the VASA survey. While one antenatal care (ANC) visit was high at 94%, the recommended four ANC visits was much lower at 41% and just 17% of the mothers had their urines tested during the pregnancy. 173 (54%) mothers of the deceased newborns had at least one labor/delivery complication that began at home. The caregivers of 65% (n = 75) of the 180 newborns that were born at home or born and left a health facility alive perceived them to be severely ill at the onset of their illness, yet only 44% (n = 80) attempted and 36% (n = 65)could reach the first health provider after an average of 91 minutes travel time. Distance, lack of transport and cost emerged as the most important constraints to formal care–seeking during delivery and during the newborn fatal illness.

**Conclusions:**

This study suggests that maternal and neonatal health organizations and the local government of Malawi should increase the demand for key maternal and child health interventions, including the recommended 4 ANC visits, and ensure urine screening for all pregnant women. Early recognition and referrals of women with obstetric complications and interventions to promote maternal recognition of neonatal illnesses and care–seeking before the child becomes severely ill are also needed to improve newborn survival in Balaka and Salima districts of Malawi.

The international community has recently published the Every Newborn Action Plan, endorsed by governments, the private sector, civil society and other stakeholders, that calls for reducing neonatal mortality rates in all countries to fewer than 10 deaths per 1000 live births by 2035 [1]. This new plan postulates that high coverage of interventions before, during and after pregnancy could save nearly 3 million women, stillbirths and newborns by 2025 in 75 high–burden countries [1].

With 68 deaths per 1000 live births in 2013, Malawi is one of the 60 countries that has had a remarkable decline in under–five mortality by at least 72% since 1990, and is among the top 10 countries with the largest declines in neonatal mortality: from 50 to 23 neonatal deaths per 1000 live births in 1990 and 2013, respectively [2]. Nevertheless, much needs to be done to achieve more. Further reductions in neonatal deaths at the country level in particular will also require better knowledge of direct causes, but also the determinants that lead to these deaths.

More recently, there has been a growing interest in the use of a social autopsy tool to explore the social, behavioral, and health system determinants of maternal, newborn, and young child deaths [3,4].

Previous research on the topic suggested that the determinants of neonatal death may be socio–environmental, behavioral or related to quality of health care [5]. Santarelli [6] explained that countries could produce the desired neonatal health outcomes if the capacities and awareness of individuals, families, and communities – in other words, the social determinants of health – are improved, and linkages between them and the health care delivery system are built and strengthened. Similarly, current international opinion suggests that both facility and community approaches are important to ensure the continuum of care throughout pregnancy, childbirth, and postpartum periods [7,8]. In addition, reviews concluded that effective interventions for maternal and neonatal health already exist, and that large reductions in mortality could be achieved by increasing their coverage [9,10].

The overall objective of this study focused solely on the social autopsy component of the data to uncover the most common household, community and health system factors that contributed to the newborns’ deaths. Specifically, the study aimed to: 1) review the coverage among deceased neonates of key and evidence–based interventions along the continuum of normal maternal and newborn care, ie, interventions that should be normally accessible to all pregnant mothers and newborns; and 2) describe breakdowns in the care provided for neonatal fatal illnesses within the Pathway to Survival framework. This information is intended to increase our understanding of the social and modifiable factors that can be addressed or reinforced to improve the design and implementation of maternal, neonatal and child health programs in Malawi.

## METHODS

### Study sites/districts and sample

The VASA interviews in Malawi were conducted regarding deaths identified by a 24 000–household survey for the Real–time Mortality Monitoring (RMM) project undertaken by the National Statistics Office and Johns Hopkins Bloomberg School of Public Health (JHU) from October 2011 to February 2012 in the districts of Balaka and Salima, in the South and Central Regions of Malawi, respectively. The survey used a full birth history interview of women 15–49 years of age to measure child mortality. Details of the RMM survey procedures are published elsewhere [11]. To limit issues related to faulty recall, while obtaining an adequate sample size, the VASA study examined deaths within a 4–year recall period. There were 537 neonatal (0–27 days of age) deaths and 1018 deaths of young children (1–59 months of age) from 2008 to 2011. In order to decrease the respondent’s burden, we selected one death per household and that procedure resulted in a sample size of 476 neonatal deaths and 819 1–59 month–old deaths (total: 1295 deaths), which provided a precision of ±7% for neonatal deaths and ±5% for young child deaths. The current paper reports on the findings for the neonatal deaths.

### Data collection tools and VASA interview

A detailed description of the tools is available in a recent study [12]. In summary, the original English version of the VASA questionnaire was translated into the Malawi local language, Chichewa, which is spoken by most persons in the study area. A local anthropologist was recruited to do the translation and another team of two experienced staff members at the Malawi National Statistical Office (NSO) independently back–translated the Chichewa questionnaire and compared this to the original English questionnaire. The discrepancies were then scrutinized to determine the source of the errors and these were corrected through consultations between the anthropologist and the back translators. Finally, the translations were inserted into a CSPro–based software application developed to enable the direct, field–based CAPI (Computer Assisted Personal Interview) capture of the VASA interview data on a netbook computer.

Data collectors were recruited based on prior experience in conducting structured interview mortality surveys, in utilizing a personal or netbook computer or Personal Digital Assistant (PDA) and any other experience in the use of electronic devices, such as smart phones for data collection. The study preferred female data collectors due to the cultural aspects of the setting, and to a lesser extent religious beliefs in the study areas, where topics related to pregnancy, caregiving, still births and child deaths are most openly discussed among the women. Data–collectors received a three–week training session.

The training focused on technical aspects as well as ethical issues in matters of sensitivity, confidentiality, and prescribed assistance to bereaved respondents. It also included three day–long visits to the field for practice during which the fieldworkers familiarized themselves with the questionnaire and the use of netbook computers in conducting interviews.

In each district, four teams of four interviewers and one supervisor were constituted for the fieldwork and two field coordinators were also recruited to conduct additional quality control of data collected and liaise the fieldwork to the study headquarters at NSO.

The interviewers were trained to select as the respondent the person most knowledgeable of the child’s fatal illness and care provided to the child for the illness. The interview covered the fatal illness from onset to death, including for neonatal deaths, the mother’s pregnancy and delivery. Hence, additional eligible respondents were permitted if necessary. In cases with discordant responses among respondents, the main respondent’s answers outweighed that of the others.

Most of the fieldwork was conducted from March 8 to April 26, 2013. The CAPI capture allows for automated implementation of skip patterns and internal consistency checks that considerably improve the quality of the interview being conducted. However, further review of the collected data revealed 172 cases with large discrepancies between the expected (from the RMM survey) and observed birth dates, ages at death and/or gender of the deceased children. Thus revisit of those households was conduct in order to resolve the discrepant cases, along with some missed and postponed interviews.

### Data analysis

The analysis of data on preventive and curative care followed the same procedures as described in a prior publication [12]. In summary, the analysis was guided by the coverage of key indicators along the continuum of normal newborn care for well children and illness recognition and care seeking for child illnesses encompassed by the Pathway to Survival model [13–15]. The study added an extended pathway for neonatal illnesses that examined the continuum of normal antenatal care and recognition of and care–seeking for maternal complications during pregnancy, labor and delivery. In order to assess the impact of perceived illness severity on caregivers’ attempts at care–seeking for their child’s illness, a scoring system was developed based on their reports of the child’s feeding behavior, activity level and mental status. Details of the method were provided in a prior paper [12].

Cronbach’s alpha coefficients [16] of the summated scores showed values of 0.93, 0.95 and 0.96 at onset of the fatal illness, when the decision to seek care was made, and after leaving the health provider, respectively. This suggested that the items in the scores elicited highly consistent responses, justifying the reliability of the summated scores according to Nunnaly criterion [17].

### Ethical considerations

Ethical clearance for the VASA study was obtained from the Johns Hopkins School of Public Health’s Institutional Review Board and the Malawi National Health and Science Research Committee. All respondents provided informed consent before the interview was conducted.

## RESULTS

The VASA study completed interviews of 399 (84%) of 476 neonatal deaths identified by the RMM survey within the 4–year recall period and selected for study by the VASA. Of these, 290 were confirmed as neonatal deaths; 34 and 75 were determined to be young child deaths and stillbirths, respectively. Inversely, the VASA interview found that 30 of the initially sampled young child deaths were neonatal deaths. In total, the VASA study in Malawi completed 320 neonatal deaths interviews that are included in the present study.

### Demographic and household characteristics

The demographic characteristics of the deceased newborns are presented in **Table 1**. The median age at illness onset was 1 day (Mean: 3.7, standard deviation (SD): 5.70). The illness of half of the newborns lasted less than a day (Median: 0 days). Seventy percent of the deaths occurred within the first week after birth. Neonatal death was more prevalent among boys than girls with a masculinity ratio of 131. The majority of births (69%) occurred at a health facility. Similarly, 59% of the newborns died at a health facility or on– route to a health facility. Of the 219 neonates born at a health facility, about 62% died at that facility, ie, they did not leave alive.

**Table1:** Characteristics of the deceased neonates

Characteristics	Frequency (%)
**Sex:**	
Boy	181 (56.7%)
Girl	139 (43.3%)
Masculinity ratio (Boy/Girl×100)	131
**Mean age at death (in days)**	5.1 (median 2; range: 0–27)
0–6	224 (70.0%)
7–27	96 (30.0%)
**Median age at illness onset**	1 (mean = 3.7; SD = 5.70)
**Median illness duration**	0 (Mean = 1.6; SD = 2.93)
**Place of birth:**	
Hospital	122 (38.3%)
Other health provider or facility	97 (30.2%)
On route to a health provider or facility	8 (2.6%)
Home	77 (24.0%)
Other	16 (5.0%)
**Place of death:**	
Hospital	121 (37.9%)
Other health provider or facility	55 (17.1%)
On route to a health provider or facility	18 (4.3%)
Home	118 (36.8%)
Other	13 (3.9%)
Child was born and died at health facility (without leaving health facility) (n = 219)	135 (61.6%)

SD – standard deviation

The characteristics of the mother, her domestic partner, and the household are shown in **Table 2**. Approximately 84% of the mothers were married or living with a man at the time of the interview, the vast majority of them (73.8%) entered in union before 20 years of age. In the survey, the majority of the mothers of the deceased newborns had little or no education. On average, the mother had 4.5 years of schooling ranging from 0 to 14 years. Hence, about a quarter (24.2%) of the mothers of the deceased newborns were illiterate or had 0–3 years of schooling. The occupation most cited for the breadwinner was farmer/agricultural worker (35.8%).

**Table 2:** Characteristics of the mother and her household

Maternal characteristics	Frequency (%)
**Married or living with a man**	268 (83.8%)
**Age when first married (years)**	18.2 (median 18; range: 12–26)
<16	22 (8.1%)
16–19	176 (65.7%)
20+	64 (23.7%)
Don’t know	7 (2.5%)
**Mother’s mean age at time of child death (in years):**	25.7 (median 24; range: 15–47)
<16	7 (2.1%)
16–19	52 (16.2%)
20–24	104 (32.4%)
25+	150 (47.0%)
Don’t know	8 (2.3%)
**Median years of maternal schooling (in years):**	4.0 (mean 4.5; range: 0–14)
0–3	139 (43.5%)
4–6	89 (28.0%)
>6	90 (28.4%)
Don’t know	1 (0.2%)
**Father years of schooling (mean years of schooling):**	6.3 (median 6; range: 0–19)
0–3	67 (24.2%)
4–6	72 (26.0%)
>6	138 (49.8%)
**Household characteristics:**	
Main breadwinner	
Father	287 (89.6%)
Mother	15 (4.8%)
Other	18 (5.6%)
Main breadwinner is farmer/agricultural worker	115 (35.8%)
Average time at current residence	13.0 (median 10; range: 0–60)
**Household size (mean):**	4.4 (median:4; range: 1–9)
Household has electricity	11 (3.3%)
Use of piped water –In–house water supply	42 (13.2%)
Use of improved sanitation (Improved pit for toilet)	24 (7.6%)
Separate room for cooking	194 (60.5%)
Household uses firewood for cooking	286 (89.5%)
Floor of the house made of cement	29 (9.0%)
Mean travel time to nearest health facility (minutes)	106 (median: 90; range: 0–420)
**Social capital:**	
In last 3 y, community worked together on at least 1 of the following: schools, health, jobs, credit, roads, public transport, water, sanitation, agriculture, justice, security, mosque/church	318 (99.4%)
Mother was NOT able to turn to any persons or community groups or organizations for help during the pregnancy or child’s fatal illness	102 (31.8%)
Mother and her family have never been denied any of the following community	312 (97.4%)

The average household size was 4.4 persons. Only 3% of the households had electricity, 13.2% had access to an improved source of drinking water, 7.6% used improved sanitation (flush or improved pit toilet), 89.5% of the households used firewood for cooking. About 9% of the households had cement flooring, and 2 in 3 (60.5%) of households had a separate room for cooking. It took 106 minutes on average for the caregiver to reach the usual health center from her household. The families had been living in the same community for about 13 years on average, yet 32% of the mothers did not have anyone to help them during their pregnancy or child’s illness.

### Maternal and newborn care

**Figure 1:**
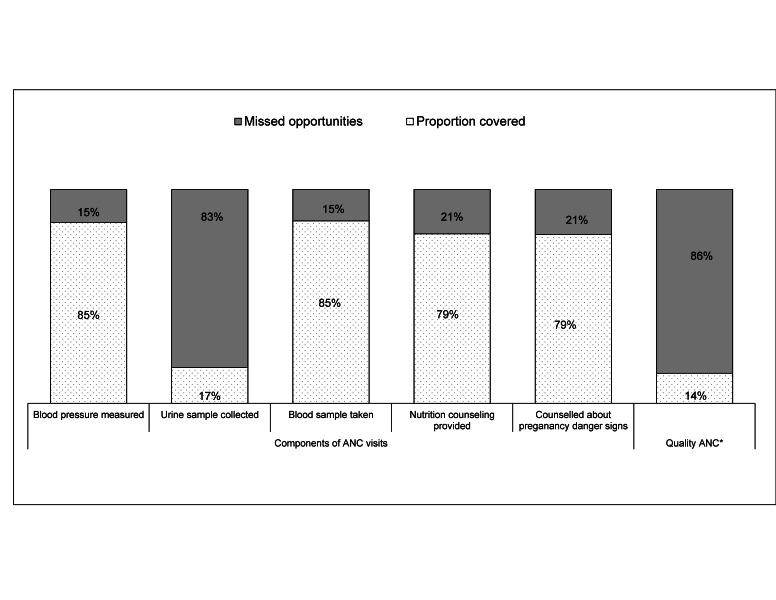
Quality gap for at least one antenatal care visit (n = 299). For women who went to at least one antenatal care (ANC) (n = 299) visit, a quality gap (or missed opportunity) exists and represents the difference between the expected maximum coverage and the actual coverage proportion. Asterisk indicates that quality ANC includes blood pressure checked, urine and blood tested, counseled about nutrition, and counseled about pregnancy danger signs.

The components of the antenatal care (ANC) received by the 93.5% (n = 299) of mothers who completed at least one visit during their pregnancy are shown in **Figure 1**. A quality gap existed because some did not receive all of the ANC components, including blood pressure measurement, urine and blood sample tests, and counseling on proper nutrition and pregnancy danger signs. In the current study, urine testing was very low (17%) among mothers of deceased newborns, and the numbers did not improve (18%) even after four or more ANC visits. Overall, just 14% of the mothers who went to a health provider for at least one ANC visit received ANC of “quality”. And the quality gap or missed opportunity ranged from 15% for blood pressure checked to 83% for urine testing.

**Figure 2:**
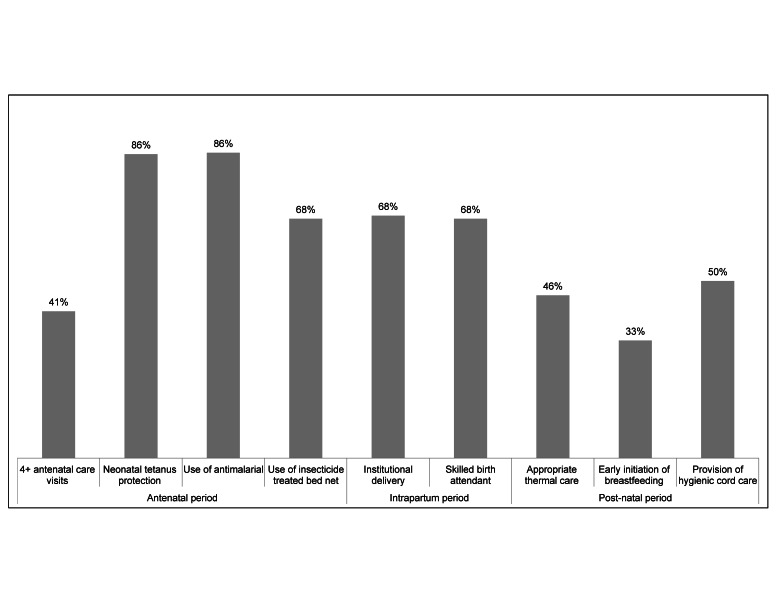
Preventive care of the mothers and newborns (n = 320).

**Figure 2** shows the preventive care received by mothers and newborns along the continuum of care. Just 41% (n = 133) of the mothers had the recommended four or more ANC visits, of which only 15% (n = 19) received ANC of “quality”. Two–thirds (68%) of the mothers delivered at a health facility, and an equal proportion were delivered by a skilled birth attendant (ie, doctors, nurses, or midwives). Among the neonates who survived the first day of life, 46% received appropriate thermal care consisting of immediate warming, drying and wiping, wrapping in a blanket, skin–to–skin contact with the mother or being placed in an incubator, plus bathing delayed for more than 24 hours after birth. Only one in three (33%) of the deceased newborns was breastfed immediately (within an hour) after birth. Overall, 67% of the newborns were first put to their mother’s breast within 24 hours. About 50% of the newborns were provided hygienic cord care, which includes using a razor blade that is new, boiled or from the delivery kit for cutting the cord, a cord clamp or thread that is new, boiled, or from the delivery kit for tying the cord and either nothing or alcohol or another antiseptic or antibiotic ointment, cream or powder being applied to the cut cord stump.

**Figure 3:**
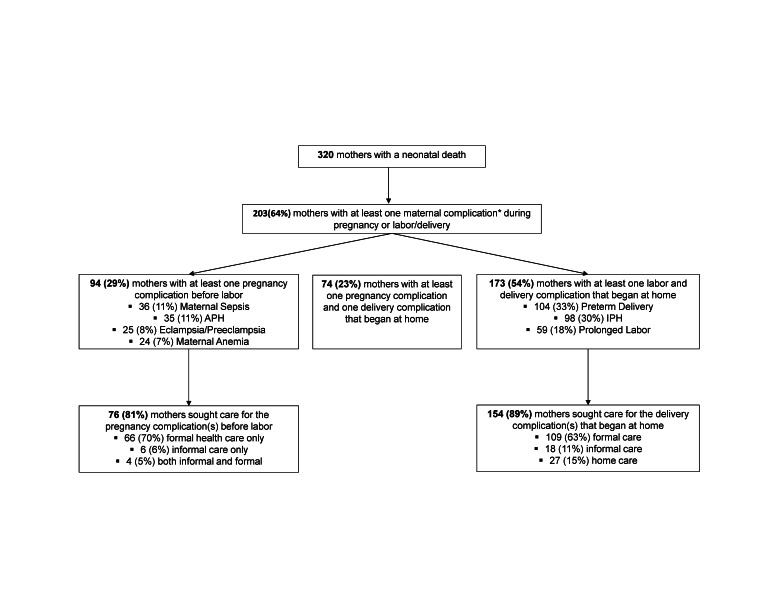
Maternal complications and care-seeking during the pregnancy and delivery (n = 320). Asterisk indicates the following: Maternal complications – Maternal sepsis = Fever+(Severe abdominal pain OR Smelly vaginal discharge); Eclampsia/ Pre-eclampsia = Severe headache+(Blurred vision OR Puffy face OR Convulsions OR High blood pressure); Maternal anemia = Severe anemia or pallor and shortness of breath+(Too weak to get out of bed OR Fast or difficult breathing); Ante–partum hemorrhage (APH) = Any bleeding before labor; Intra-partum hemorrhage (IPH) = Excessive bleeding during labor or delivery.

The maternal complications and care–seeking for these during the pregnancy and/or delivery are presented in **Figure 3**. Of the 320 mothers of deceased newborns, 94 (29%) reported they had one or more pregnancy complication(s) during the last three months, mainly maternal sepsis (n = 36, or 11%) and antepartum hemorrhage (n = 35, or 11%). More than half (n = 173, or 54%) of the mothers had at least one labor/delivery complication that began at home, including preterm delivery (n = 104, or 33%), intra–partum hemorrhage (n = 98, or 30%), and prolonged labor (n = 59, or 18%). Overall, 75% (n = 70) of the 94 mothers with a pregnancy complication sought some formal care for the complication. Several (n = 109, 63%) of the 173 mothers with at least one labor or delivery complication that began at home sought some formal care.

### Care–seeking for newborn’s fatal illness

**Figure 4:**
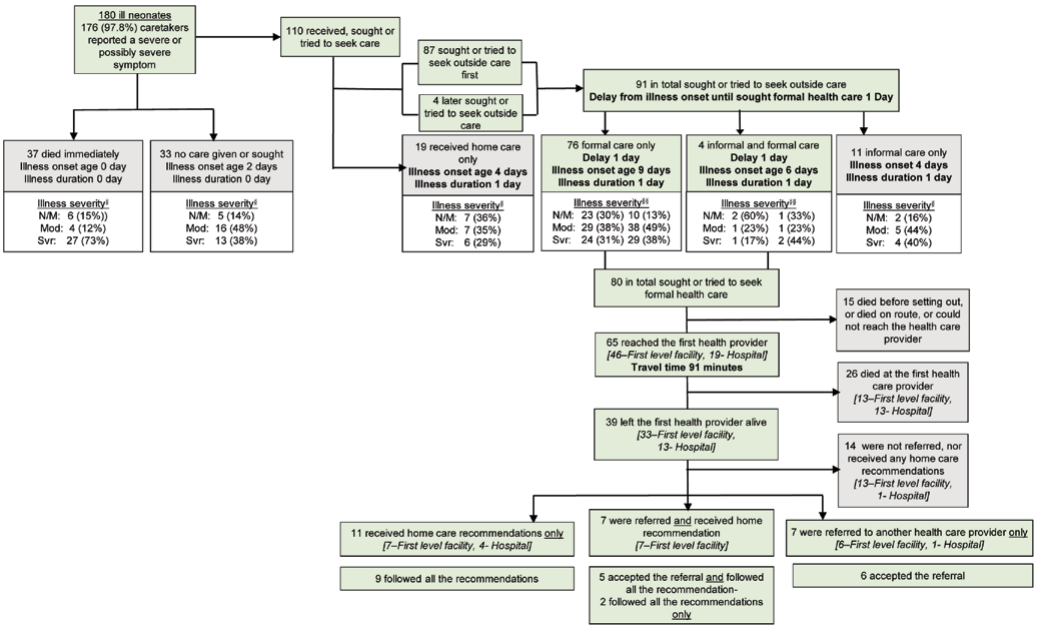
The “Pathway to Survival” for 180 neonatal deaths (born at home or left the delivery facility alive), Malawi 2008-2011. Notes: ^§^Illness severity at onset; ^§§^Illness severity at onset and when caregiver decided to seek formal care; N/M – normal/mild, Mod – moderate, Svr – severe.

The breakdowns in the Pathway to Survival that contributed to the newborn deaths are presented in **Figure 4** for the 180 newborns who were either born at home or left the delivery facility alive and had information on care–seeking. Another 135 of the 320 newborns were born and died at the health facility without leaving, and five others had an illness that began at the facility where they were born and died later at home, but data these cases had no information on care–seeking.

When the 180 newborns’ fatal illnesses began, 97.8% of care–takers could recognize and report a severe or possibly severe symptom. Yet, the mothers of only 110 (61.1%) of the newborns sought or tried to seek care; 37 (20.6%) of the newborns “died immediately” and no care was given or sought for the other 33 (18.3%) newborns. And, 73% and 38%, respectively, of the neonates in the last two groups were ranked as being severely ill at the time their caregivers first noticed the illness. Understandably, for the group that died “immediately,” both the median age at illness onset and the median illness duration were 0 days, meaning the newborns were born and died quickly (or “immediately”) on the day of birth. For the “no care given or sought” group, the illness occurred on the third day of life (day 2) and lasted 0 days as well.

Regarding the group of the mothers of the 110 neonates who received, sought, or tried to seek care, the vast majority (79%, n = 87) first sought care outside the home, 23 first received care inside the home, and 4 of these 23 later sought or tried to seek outside care. A total of 91 sought, or tried to seek care outside the home, and the median length (or delay) from illness onset until formal health care seeking was 1 day.

Among those who sought outside care, the majority (83.5%, or n = 76) sought formal care only, 4 sought both informal and formal care, and 11 informal care only. The delay to seeking formal care was 1 day, both for those who sought informal and formal care and those who sought only formal care.

Out of the 80 newborns for whom formal care was sought, 31 (38.8%) were already severely ill at the time the caregiver decided to seek care. Fifteen (or 18.8%) did not reach the health facility because they died before setting out, died on–route or could not reach the health provider. The remaining 65 (81.3%) newborns could reach the first health provider, on average, after 91 minutes travel time. More than two–thirds (n = 46, 70.8%) of these 65 newborns were taken to an NGO or government clinic or facility led by Health Surveillance Assistants (hereafter referred to as a “first level facility),” and the other 19 (29.2%) were taken to a non–governmental organization (NGO) or a government hospital.

Twenty–six (40%) of the 65 neonates that reached a first provider died at that provider, including the 13 out of 19 (68%) that reached a hospital. Yet, 14 of the 39 (36%) who left the first health provider alive were not referred nor received any home care recommendations. However, when recommendations were received, or referral provided, most of the caregivers (80–100%) followed all the recommendations or accepted the referral and went to a second health care provider.

### Maternal and newborn care–seeking constraining factors

**Figure 5:**
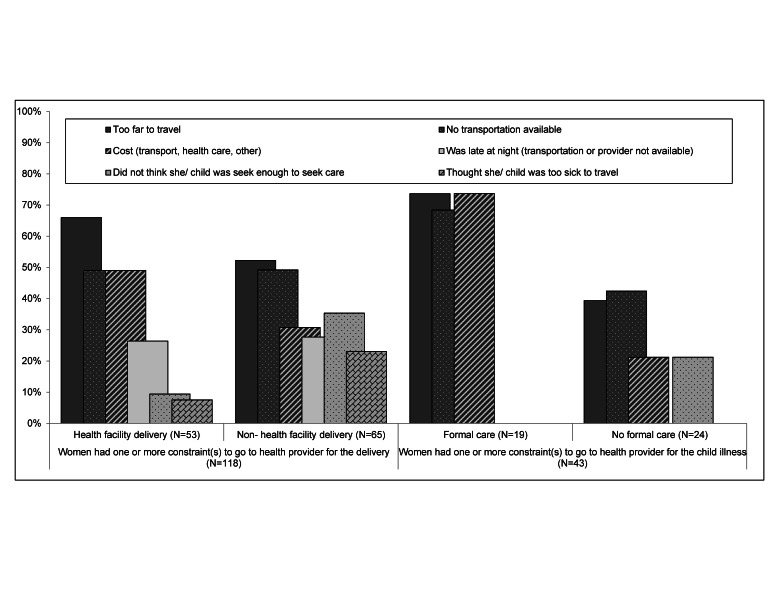
Main care–seeking constraints for the delivery and for the neonatal illness.

The care–seeking constraints for the delivery and for treatment of the neonatal illness are described in **Figure 5**. In total, 118 mothers reported concerns or problems regarding delivering at a health facility, of which 53 succeeded in doing so. In other words, of the 219 mothers who delivered at a health facility, 53 (24.1%) had to overcome some concerns or constraints to deliver at the facility. Conversely, the vast majority (64%, n = 65) of the 101 mothers who did not deliver at a health facility reported constraints or concerns in doing so. And the difference between the two proportions (24.1% vs 64% was statistically significant (*P* < 0.001).

Of the 143 neonates that sought/tried to seek care or not (ie, the groups of 33 “no care was given or sought” and 110 “received, sought or tried to seek care”) for the fatal illness, the proportion of those who reported any constraints was statistically higher in the subgroup that either did not seek care or sought home or informal care only (n = 63) than in the subgroup that sought some formal care (n = 80) (54.1% vs 23.6%, *P* < 0.001).

Among the care–seeking constraints reported either during delivery or during the newborn fatal illness (**Figure 5**), the following emerged as most important: distance (52–74%), lack of transport (49–68%), and cost (21–74%).

## DISCUSSION

The objective of this social autopsy study was to shed light on the social, behavioral, and health system determinants of newborn deaths from 2008 to 2011 in Balaka and Salima districts in Malawi.

### Demographic and household characteristics

Our study showed that the majority of the deceased newborns were from households with poor socioeconomic conditions, lacking basic commodities such as electricity, water sanitation and clean water. The households were crowded and about 90% used firewood for cooking. The breadwinners were mostly farmers. These hardship living conditions have been shown to increase the risk of illness for mothers and newborns because they face more challenges in accessing timely, high–quality care compared to wealthier families [18].

The gender differential in neonatal mortality is also worth mentioning. It is well known that more boys than girls are born globally, but boys are more likely to die than girls in the neonatal period mainly due to higher vulnerability to infectious diseases [19]. Likewise, in this study, the number of newborn deaths was 1.3 times greater among male than female children.

### Maternal complications and care during pregnancy, labor and delivery

Recent reports from Malawi have shown improvement in perinatal and neonatal outcomes and an increased coverage by health services and skilled birth attendants across the country [2,20]. Our study found that for mothers with a neonatal death, coverage of at least one antenatal visit was relatively high at 94% in these two Malawi districts. Yet coverage of the recommended minimum four visits (41%) was much lower. It is well known that certain interventions such as iron and folic acid, antimalarial drugs prophylaxis, syphilis testing and treatment, and tetanus toxoid immunization cannot be effectively delivered with only one antenatal visit. Thus, if antenatal care is to contribute to reducing the neonatal mortality rate in the study setting, a minimum of four antenatal care visits with the full range of evidence–based interventions at each visit is required [21].

The quality gap found in our study implies that 86% of mothers with a neonatal death did not receive the quality of antenatal care that they needed—even when they had contact with the health system during their pregnancy. This quality gap persisted even with an increased number of ANC visits. Without improved quality, increased antenatal coverage is unlikely to substantially improve perinatal and neonatal outcomes [22,23]. For instance, during the antenatal visit(s) the vast majority of mothers did not have their urine tested during the course of their pregnancy. Similarly, according to a 2010 emergency obstetric and newborn care (EmONC) facility–based survey [24] and the most recent Malawi Service Provision Assessment [25], very few facilities offering ANC services in the country have the capacity to conduct laboratory diagnostic tests due to lack of equipment, including urine chemistry testing. And Government facilities were least likely to have dip sticks for urine protein and glucose (12% and 9%, respectively) compared to facilities managed by other authorities [25]. This appears to be a problematic situation. A urine test should be conducted during the first prenatal exam and then at least periodically in future prenatal visits. Urinalysis is used to assess bladder or kidney infections, diabetes, dehydration, and preeclampsia by screening for high levels of sugar, protein, ketones, and bacteria [26, 27]. Higher levels of protein may suggest a possible urinary tract infection (UTI), or kidney disease. It is well established that during pregnancy, UTI is associated with increased risks of preterm delivery, even when the infection is asymptomatic [28]. The present study found that preterm delivery was the main complication experienced by the mothers during delivery, which coincides with that of “Born Too Soon” reports in which Malawi has the highest rate of preterm birth in the world [29].

The dearth of available information on UTI rates in Malawi [28], added to the low proportion of urine testing among mothers as shown by the current study could reflect the overall lack of health care infrastructure for research, screening, and treatment programs in Malawi. A study design that would investigate the prevalence of UTI among women with preterm delivery complications in the country would be of importance since that could fuel policy makers’ interest and justify the promotion of universal modern antenatal urine screening, combined with appropriate follow–up management or treatment of UTI, either symptomatic or asymptomatic. Elsewhere [28], screening and treatment of asymptomatic bacteriuria (ASB) has improved preterm birth and low birth weight outcomes in several developed countries and would likely improve maternal and neonatal health worldwide, particularly in developing countries such as Malawi. ASB screening has been thus included in the WHO ANC package. Darmstadt et al. estimated a reduction of prematurity and low birth weight by 20–55% and neonatal mortality due to preterm birth by 5–14% [9].

The fact that the vast majority of women received at least one antenatal care visit offers an opportunity to effectively implement the ASB screening. We acknowledge that one of the main barriers to ASB screening resides in the non–existence of adequate clinical microbiology resources in these settings. Some authors have suggested the need for innovative alternatives such as portable ASB screening devices or the need for simple methods that could be used by community health workers outside traditional clinic settings [28].

The majority (63%) of mothers who experienced intrapartum complications that started at home sought formal care. In addition, the majority of newborns (59%) born at a health facility did not leave the facility alive. These findings suggest that these facility deliveries included high–risk or complicated cases with a higher risk of early neonatal death, a situation that has been observed in other studies [30,31]. The quality of care in the facilities cannot be assessed by this study but must be examined to determine if more of these deaths could have been prevented.

In addition, the majority of mothers whose newborns were born and died in a health facility without leaving had one or more labor or delivery complications that started at home. The vast majority of intrapartum events—such as heavy vaginal bleeding, preterm delivery and prolonged labor—have been described as significant risk factors for early neonatal deaths [32,33]. Therefore, early recognition, immediate care–seeking and referral (when needed) of women with obstetric complications to an appropriate center should be an intervention program priority, including essential and emergency obstetric and newborn care and newborn resuscitation services [34].

### Care–seeking for newborn’s fatal illness

Of the 180 newborns whose fatal illness started at home, almost all the caretakers (97.8%) recognized a severe or possibly severe symptom. Yet only 80 sought or tried to seek formal care, of which the proportion of the perceived severely ill newborns increased from 31% to 39% from onset of the illness to the moment that caregivers decided to seek formal care. Yet just 65 (36%) could reach the first health provider after an average 91–minute travel time. This finding suggests that there was a delay in recognizing an illness of severity that should have led to prompt care–seeking. In addition, caregivers had to overcome the constraining travel distance to reach the health facility. Timing is important for providing neonates with appropriate and prompt care at the onset of illness, and any delays in the decision to seek care, or in taking the action of care–seeking can be fatal to the infant [35]. Yet, prior to the decision to seek care, caregivers need to be able to recognize the illness danger signs of their newborns. This awareness can be particularly challenging in the neonate due to the lack of specific symptoms [36–38]. Care–seeking was also delayed for several neonates who became sick after the first week of life and whose illnesses were less serious at the onset until they became more severely ill. Other studies have described interventions to promote maternal recognition of neonatal illnesses and care–seeking before the child becomes severely ill [39–41].

Our study also suggests that referral and home care recommendations for sick newborns need to be improved at least among first level health facilities, as findings show that more than a third (13 newborns out of 33 that reached and left a first level facility) of the of sick newborns were not referred nor received any home care recommendations.

### Care–seeking constraining factors

Our findings further show that distance, lack of transport and unaffordable costs, including transportation and health care costs, emerged as the most important constraints, both for any pregnancy or labor/delivery complications and for newborn fatal illness. The Ministry of Health estimated that only 54% of the population has access to a health facility within a 5 km radius [42]. These barriers suggest that women from the most remote areas are still at a disadvantage because they have the longest distance to travel. They may also deliver on–route to the health facility, where they are arguably worse off than if they had delivered at home. They may only set out when their child’s severe illness has already started. Powell et al. [43] described a financing scheme in Nepal that provided transport costs to further reduce the barriers to health care for the poorest women, but this was challenging to implement. Another example includes the implementation of an obstetric helpline and taxi transport schemes in response to the financial and distance barriers identified by the Maternal and Perinatal Death Inquiry and Response (MAPEDIR) project in Dholpur district of Rajasthan State in India [44].

### Limitations

The limitations of this study have been also discussed elsewhere [12].The data could have been affected by recall bias and the possibility of providing socially desirable answers to sensitive questions, given the recall period of about 4 years, in addition to the fact that the respondents were the main caregivers of the deceased child. However, we believe that the conversational and prompting modes used during the face–to–face interviews and the involvement of experienced interviewers may have led to better overall recall of events. In addition, the study was conducted in just two districts in Malawi; hence readers should exercise caution in the attempt to generalize the findings to the entire country situation. A national and representative sample VASA study design could offer a clearer picture of the entire country. The inclusion of a control group would have allowed the analysis to test whether there were significant differences between the coverage of interventions among cases (deceased newborns) and controls (alive newborns); however, the lack of a comparison group in SA studies is common and not so necessary since we were studying interventions that should be accessible to all pregnant mothers and newborns and that had been proven to be effective against neonatal mortality [15].

## CONCLUSIONS

Encouragingly, Malawi appears to have made great strides recently in achieving declines, albeit slow, in neonatal mortality rates. The current study went beyond the classic review of interventions for deceased newborns and their mothers along the continuum of care, to identify the breakdowns within the Pathway to Survival that led to newborn deaths and to examine the care–seeking barriers for maternal complications during pregnancy and delivery, and the newborns’ fatal illnesses that contributed to the deaths. The ultimate goal of this study was to increase our knowledge of modifiable factors that could be targeted in order to improve neonatal health for progress in an overall more rapid decline in neonatal mortality in Malawi. Hence, our main results confirmed significant progress in some areas of women’s and children’s health, such as facility delivery and skilled birth attendance and attendance of mothers to at least one antenatal visit. Yet, increasing the number of pregnant women who obtain antenatal urine testing and treatment and who attend at least 4 ANC visits as recommended by WHO could be a worthwhile approach for improving birth outcomes. In addition, early recognition and referral of women with obstetric complications and interventions to promote maternal recognition of neonatal illnesses and care–seeking before the child becomes severely ill are needed to accelerate the decline in neonatal deaths in the study districts.
